# COVID-19 Management at IHU Méditerranée Infection: A One-Year Experience

**DOI:** 10.3390/jcm10132881

**Published:** 2021-06-29

**Authors:** Philippe Brouqui, Michel Drancourt, Didier Raoult, on behalf of the IHU Task Force

**Affiliations:** Aix-Marseille University, IRD, MEPHI, IHU Méditerranée Infection, 13005 Marseille, France; philippe.brouqui@univ-amu.fr (P.B.); michel.drancourt@univ-amu.fr (M.D.)

**Keywords:** pandemic, preparedness, adaptation, autonomy in decision-making, leadership, rerouting of tests, drug repurposing, unrestricted access to care

## Abstract

Background: The Hospital-University Institute (IHU) Méditerranée Infection features a 27,000 square meter building hosting 700 employees and 75 hospitalized patients in the center of Marseille, France. Method: Previous preparedness in contagious disease management allowed the IHU to manage the COVID-19 outbreak by continuing adaptation for optimal diagnosis, care and outcome. We report here the output of this management. Results: From 5 March 2020, and 26 April 2021, 608,313 PCR tests were provided for 424,919 patients and 44,089 returned positive. A total of 23,390 patients with COVID-19 were followed at IHU with an overall case fatality ratio of 1.7%. Of them 20,270 were followed as outpatients with an overall CFR of 0.17%. We performed 24,807 EKG, 5759 low dose CT Scanner, and 18,344 serology. Of the 7643 nasopharyngeal samples inoculated in cell cultures 3317 (43.3%) yielded SARS-Cov-2 isolates. Finally, 7370 SARS-Cov-2 genomes were analyzed, allowing description of the first genetic variants and their implication in the epidemiologic curves. Continuous clinical care quality evaluation provided the opportunity for 155 publications allowing a better understanding of the disease and improvement of care and 132 videos posted on the IHU Facebook network, totaling 60 million views and 390,000 followers, and dealing with COVID-19, outbreaks, epistemology, and ethics in medicine. Conclusions: During this epidemic, IHU Méditerranée Infection played the role for which it has been created; useful clinical research to guarantee a high-quality diagnostic and care for patient and a recognized expertise.

## 1. Introduction

The very first case of COVID-19 in Marseille was diagnosed in the IHU Méditerranée Infection (IHU) in Marseille, France on February 27, 2020, and the IHU had to continuously adapt its strategy over 9 months of the epidemic in Marseille to cope with the overwhelming waves of COVID-19, later proved to be caused by at least three different lineages of SARS-CoV-2 [1,2]. We here review the key steps in this adaptation, while point-of-care (POC) laboratories will be presented in another paper [3].

## 2. Methods

### 2.1. The IHU Méditerranée Infection Built to Confront Epidemics

The IHU Méditerranée Infection (herein designated as IHU), held by a foundation called Fondation Méditerranée Infection, whose overall structure and functional organigram are presented in supplementary data, was created in 2011 as part of the program Investissements d’Avenir launched by the French government, and was the only such institute devoted to infectious and tropical diseases [4,5]. While the IHU is conveniently located in the university hospital medical campus in the heart of Marseille, it primarily serves the population of the Marseille area and Provence at large, acting as the National Reference Center for infectious diseases, hosting patients from all over France and abroad. As a private law regime foundation, the IHU has great agility in decision-making, which has been a key point in effectively confronting the COVID-19 outbreak. All strategic decisions could be validated in a weekly Director Committee meeting, decisions being immediately enforced.

The IHU is a 27,000 square meter building conceived to accommodate contagious patients and potentially hazardous samples even in the case of epidemics, consisting of four horizontal lobbies squared into three vertical sectors consisting of a university hospital sector, a laboratory sector and a tertiary sector; the overall building is under strict access control, including biometric access control in some of the more critical sectors (https://www.mediterranee-infection.com/en/, accessed date is 28 June 2021). The project consisted in building ex nihilo on the Marseille Medical Timone Campus a research hospital specifically dedicated to infectious diseases and securely protected to care for contagious patients, with a formidably equipped diagnostic microbiology laboratory capable of containing extremely contagious pathogens, including potential agents of bioterrorism, in a 1200 square meter biosafety level 3 (BSL3) laboratory. There are four research units, as well as startups and spinoffs benefiting from IHU know-how and developments in the field of infectious disease. For patient care, three wards of 25 single rooms consisting of: one ward for acute emergency infectious diseases, one ward for chronic infections and one ward dedicated to contagious diseases equipped for biosafety level 3, with 3 modules of 7, 8 and 10 beds. Module A (7 beds) is pre-equipped for intensive care, and every room is accordingly remotely controlled. A BSL3 point-of-care (POC) laboratory is located within the ward for diagnostic and routine laboratory tests for contagious patients, under the supervision of the microbiology team. Visitors are not admitted to the unit and patient visits must be accessed via an external corridor, if allowed [6]. The outpatient clinic on the first floor includes a 21-bed day hospital and a specific and dedicated area for rapid diagnostic screening, avoiding encounters between contagious patients and other patients and personnel in the institute [6]. All care facilities can be depressurized. Heath care personnel are regularly trained, based on monthly exercises and by regular real-life practice, as was the case with patients returning from Saudi Arabia and suspected of MERS-Cov [7], and they benefit from a specific medical program of vaccination-based protection against infectious diseases and serological monitoring on a voluntary basis.

In close connection with the research hospital sector, the Microbiology Laboratory of the IHU consists of POC laboratories, presented in an additional paper [3], and a large, 2341 square meter diagnostic core laboratory organized in platforms: a reception platform entering information in the laboratory informatics system, dispatching clinical sample aliquots on the technical platforms and preparing biobanking; a culture platform; a molecular biology platform performing nucleic acid extraction and PCR-based tests; a serology platform performing enzyme-linked immunosorbent assays (ELISAs), indirect immunofluorescence assays and automated Western-immunoblotting assays; and a large secured biobank with a storage capacity of 1 million samples at −80 °C and 2 million samples at −20 °C for preservation of clinical samples, isolates and nucleic acid extracts. All platforms are informatically interconnected (on-going) and informatically connected to the information system of the public university hospitals in Marseille (Assistance Publique à Marseille). In fact, platform equipment has been regularly updated in order to be permanently equipped with the most efficient, advanced diagnostic techniques. For direct examination of samples, the IHU laboratory is equipped with the latest generation electron microscopes (TM4000plus scanning electron microscope, Hitachi, Tokyo, Japan) which combine the power of traditional electron microscopes with the ease of optical microscopes, rendering electron microscopy a routine technique for the observation of samples and microbes [8]. The culture platform is equipped with the largest worldwide capacity for matrix-assisted laser desorption/ionization-time of flight (MALDI-TOF), with 8 MALDI-TOF instruments and a unique spectrum database to identify microorganisms and pathogens [9] and their potential vectors [10]. The molecular biology platform comprises 28 thermocyclers, while downstream routine sequencing was launched in 1992, after the IHU ancestor bought the first automatic sequencer in Europe. The sequencing platform is now equipped with 4 MiSeq instruments (Illumina, Paris, France), 2 Gridion and 1 PromethION instruments (Oxford Nanopore, Oxford, UK), as well as 1 iSeq (Illumina) and 4 MiNion instruments (Oxford Nanopore), more specifically dedicated to POC applications. In addition, a 1200 square meter NSB3 security laboratory with biometric access control allows for isolation, culture, manipulation and testing and storage of contagious pathogens [11]. These platforms, dedicated to the routine diagnosis of infectious and tropical diseases, are routinely served by 215 qualified personnel, including 29 certified biologists, 24 engineers and 21 residents in medical biology. All these resources have been mobilized to fight the COVID-19 epidemic in France.

### 2.2. IHU Fighting the COVID-19 Epidemic

As early as January 31, 2020, Europeans repatriated from Wuhan, placed in provisional quarantine 20 km from the IHU, were evaluated by the IHU, following its capability in developing RT-PCR testing from scratch before any diagnostic test was commercially available. Internal expertise was mobilized in primer design, based on SARS-CoV-2 viral sequence analysis and experimental protocol design, so that testing capability reached 500 tests/day for the diagnosis and follow-up during the quarantine of those repatriated [12]. A daily COVID-19 steering committee met as early as January 31 and continued as such for precise day-by-day management of the outbreak. Accordingly, we developed a rapid virological screening circuit, so that RT-PCR results were available within 3 h of laboratory management [13]. This organization plan was set up very early, just as we diagnosed the first positive patient, and was adapted throughout the outbreak to respond to changing situations, creating five different COVID-19 laboratory circuits: (1) a POC circuit, including an innovative check-point as detailed in [3,14]; (2) a hospitalized-patient circuit, with the goal of obtaining RT-PCR results before 10 a.m. in order to manage hospitalization turnover; (3) an emergency circuit, with the goal of delivering RT-PCR results within 4 h; (4) a routine circuit, with the goal of delivering RT-PCR results within 8 h; (5) and an external sample circuit, with the goal of delivering RT-PCR results within 24 h. In order to achieve these goals, we progressively increased the capacity for obtaining nasopharyngeal samples by ultimately creating five posts, using the national SI-DEP system, served by a pool of 14 recruited personnel. Additionally, core laboratory facilities were extended to three additional laboratory rooms previously devoted to research activities, adding 184 square meters of laboratory where additional instruments were installed: the Molecular Biology platform had an increase of four nucleic acid extractors for a total of 20 extractors, one RT-PCR thermocycler for a total of 19 thermocyclers, and was equipped with two plaque preparators that were not available before the COVID-19 epidemic. In addition, we added 6 informatics posts. More than 600,000 RT-PCR custom-made tests have been fabricated in the IHU. In parallel, the activity of the Biosafety Safety Level 3 laboratory was redirected towards the high throughput isolation and culture of SARS-CoV-2 strains, the majority from nasopharyngeal swabs used in parallel for RT-PCR diagnosis [15]. This activity was rapidly crucial in determining a cut-off value for the accurate interpretation of RT-PCR cycle threshold (CT) after we showed that a CT value of >34 allowed only 1% viable viruses [16]. Further, continuous high throughput isolation of SARS-CoV-2 strains proved determinant for *in cellulo* testing of the activity of different drugs, chiefly hydroxychloroquine, azithromycin and zinc [17,18,19] and the observation of SARS-CoV-2 strains exhibiting decreased *in cellulo* susceptibility [20]. Finally, SARS-CoV-2 culturing supported monitoring of partial and entire whole genome sequences to determine the various SARS-CoV-2 genotypes underlying the dynamics of COVID-19 epidemics, including the geographical sources, and provided antigens for the home-made serological tests, including indirect immunofluorescence and automated Western immunoblotting. In fact, the IHU developed from scratch SARS-CoV-2 serology based on indirect immunofluorescence before any serology test was commercially available [21]. Building such a test from scratch was made possible thanks to previous expertise acquired over the years in that technique, previously applied, among other applications, to facultative intracellular pathogens [22]. Conversely, setting-up indirect immunofluorescence SARS-CoV-2 serology provided the opportunity to automatize basically manual indirect immunofluorescence, by automatization of antigen spotting on slides (Echo 525, Labcyte, Beckman Coulter, Indianapolis, USA) and automation of slide reading using an automated fluorescent slide scanner (AxioScan Z1., Zeiss, Marly le Roi, France). All the different commercially available serology techniques were progressively adopted, including POC lateral flow assays [23], enzyme-linked immunosorbent assays (ELISA) and chemiluminescence assays [23]. In order to support the routine medical care of COVID-19 patients, the laboratory increased its capacity for hydroxychloroquine assays by liquid chromatography (LC-UV) and implemented from scratch azithromycin assays by liquid chromatography-mass spectrometry (LC-MS) (Chabrière E. et al., unpublished data). Additionally, zinc assays were monitored and the total lymphocyte count, differential CD4/CD8 and NK counts were also routinely monitored, using flow cytometry (Aquios Tetra, Beckman Coulter). Altogether, a total of 58 different personnel were recruited specifically to deal with the additional laboratory activity, including 44 laboratory technicians and 14 secretaries, a 27% increase in laboratory personnel.

**Figure 1:**
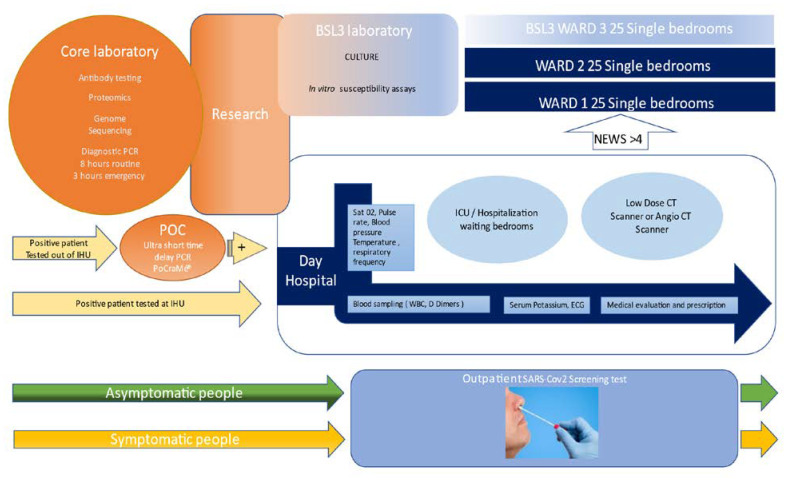
IHU organization pathway for testing and microbiology analysis, care, and research on outbreaks using the example of COVID-19, March 2020–January 2021. Research is at the interface of the laboratory, divided into a Point of Care (POC) and core laboratory; treatment, divided between the outpatient clinic and 75 bed wards, and BSL3 activities. Patient entry on the left and exit right.

For patient care, the building was separated in three parts: one dedicated to patient screening, one for ambulatory care and one for hospitalization, including 25 single rooms in biosafety level 3 and 50 single rooms in uncontrolled air depression as cited above. We first used the 25 contagion rooms of the dedicated BSL3 ward, which gave us time to reorganize the other two wards. This time was also precious for our university hospital (AP-HM), as it provided three additional weeks to organize the surge capacity for the care of COVID patients concentrated in our institute during this time. Once the 75 beds were full, we organized the turnover of contagious patients (see below). Because symptomatic people were more likely to be PCR positive, the screening circuits were organized in two lines: symptomatic and asymptomatic people were separated (Figure 1). The continuously rising number of people that came for testing during the first wave (peak of 3596 tests on 3 April 2020) and the prolonged waiting time in the line (up to 3 h), with people arguing, became a true problem, and led us to set up a specific COVID 19 testing plan from 7:00 a.m./7:00 p.m., 6/7 days by individual appointment using the commercially available French web application “Doctolib,” or without appointment in dedicated time slots. This organization was so effective that we were able to test a thousand people per day without further trouble. Nasopharyngeal sampling was carried out by trained nurses and the samples were transferred immediately to the laboratory. Patient registration, presentation (symptomatic or not), and PCR results, ratio of infected/uninfected and ratio of positive in symptomatic and asymptomatic individuals was available in real time, 24/24 h 7/7, on a dedicated screen and was used to monitor the epidemic. The standard turnaround time for PCR test results was 8 h for routine diagnosis and 3 h for the ICU and emergency department. We automatically sent all positive patients a brief text message, asking them if they wished to volunteer to be treated and followed in our center. All positive PCR tests performed elsewhere than the IHU where subject to control, with an ultra-short PCR testing turnaround available in 20 min, before enrolling the patient for care, which is reported in more detail in this issue [3].

The day hospital was organized to screen patients by nurses, with monitoring of vital signs, including pulse oximetry and laboratory investigation (D-dimers, C-reactive protein, fibrinogen, white blood cells and eosinophils), and an electrocardiogram was performed in all patients; abnormal EKGs were remotely monitored by the cardiology department. The day hospital was organized to screen ambulatory patients. The vital signs, including pulse-oximetry and laboratory investigation (D-Dimers, C reactive Protein, fibrinogen, WBC and eosinophils) were carried out by nurses, electrocardiogram was performed to all patients and abnormal ones were controlled by tele consultation with the department of cardiology. Serum potassium levels were obtained in real time at the point of care (i-STAT ALINITY, Abbott Point of Care Inc, Princeton, NJ, USA.) to eliminate delays in treatment with HCQ and AZT. A low-dose CT lung scan was systematically carried out in patients older than 55 and/or with comorbidities and/or lung abnormalities on clinical examination and/or a SaO2 < 95%. The low-dose CT scans were conducted in the radiology department [24,25]. Medical doctors established the prognosis using the News 2 score and evaluated the need for transfer to hospitalization (News > 4) or intensive care. Patients with abnormal ECGs or QTc > 460 ms and/or K < 3.6 mmol/l and/or receiving drugs not compatible with HCQ were contraindicated for HCQ and treated with AZT/Zinc. The remaining patients were asked if they wished to be treated with the combination of HCQ/AZT/Zinc. Outpatients with a comorbidity or older than 55 were advised to monitor their oxygen saturation at home, even if they felt comfortable, and to consult their doctor immediately if the Sa02 <95% on two occasions [26]. In patients with a risk of thrombosis we prescribed anticoagulant therapy. When the D dimers were above 0.5 µg/mL a doctor called the patient back and prescribed anticoagulant therapy, and if >2 µg/mL the patient was asked to present immediately for a CT angiogram to rule out pulmonary embolism. All patients were systematically told to come back to the hospital in case of need. All patients were asked to come back at day 10 for PCR testing [27].

The same protocol was applied to patients treated in the IHU wards. All patients were offered HCQ/AZT/Zinc if they volunteered and had no contraindications [28,29]. The treatment protocol was adapted during the progression of the outbreak with the addition of anticoagulant therapy, steroids (dexamethasone 6 mg/d) for severely ill patients requiring oxygen and in the inflammatory phase of the disease when the CT indicated that the viral load was either negative or very low, and finally with high flow oxygen therapy in patients for whom the intensive care unit was not indicated [30]. To accelerate patient rotation and enhance the capacity of the IHU to isolate contagious COVID-19 patients, we transferred all patients exhibiting 2 negative SARS-CoV-2 PCR, defined as a CT >34 based upon our own laboratory data, to other units [31]. Every morning a general staff meeting was held to adjust patient care (see above).

## 3. Results

**Figure 2:**
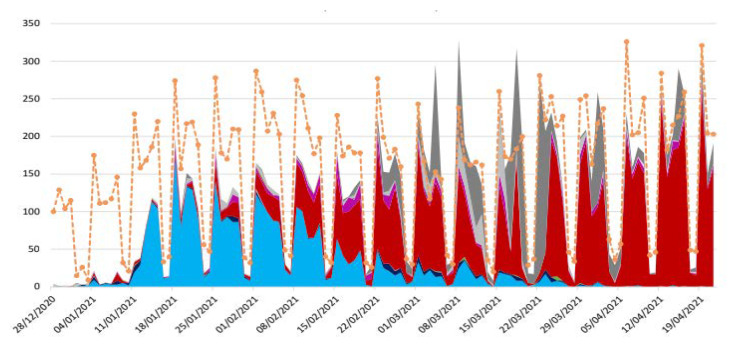
Distribution of SARS CoV 2 variant during the epidemic in Marseille France. Orange = total positive test, electric blue = Marseille 4 variant (20 A.EU2), red = UK variant, deep pink = South African variant, pink = Brazil variant. To note that the proportion of genome sequenced virus grew quicky 22 February 2021.

**Figure 3:**
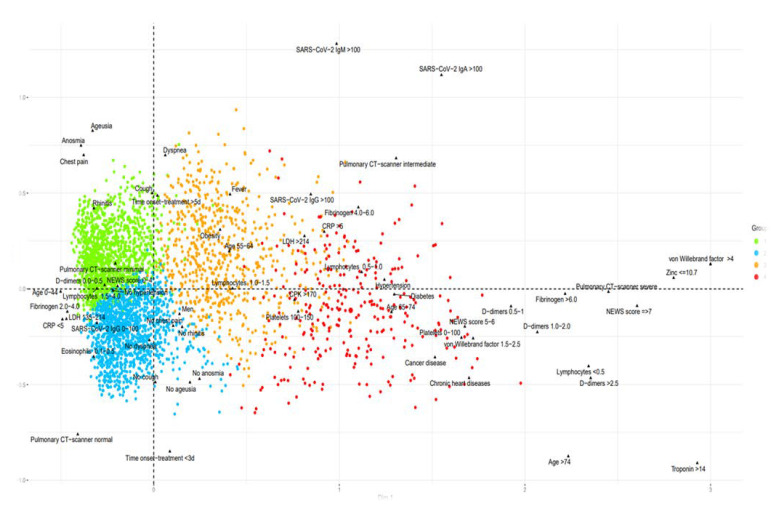
Multiple correspondence analysis (MCA) including all the clinical and biological radiological data and the outcomes of the COVID patient cohort of the IHU. Clinical expression of COVID-19. In green upper respiratory COVID-19 with anosmia and ageusia, in blue asymptomatic hypoxemia, in orange COVID-19 pneumonia, and red severe COVID-19.

From 5 March 2020 to 26 April 2021 at the IHU, we conducted 608,313 SARS-CoV-2 PCR tests for patients in our institution (AP-HM, including IHU) and for people coming from everywhere in the French territory. Of them 204,982 attends directly IHU to be tested as reported above. Of the 424,919 patients tested at our institution, 44,089 (10.3%) were positive for SARSCOV 2; of them, 23,390 were cared at the IHU; 20,270 at the day hospital and 3120 in the 75 bed of the institute. 19,543 patients were cared at the nearby university Hospital (APHM) and 1244 elsewhere. The case fatality ratio (CFR) was 1.70, 4.04, 4.41 when patients were cared at IHU, APHM, or elsewhere respectively (OR 0.41 CI 95% 0.35–0.47 *p* < 0.001). For patients cared as outpatient at IHU 36/20.270 died (CFR 0.17%). We conducted 5759 low-dose CT scans of the lung and 24,807 ECGs. The radiology department was situated 15 m a part of the IHU building. Patients were transferred with PPE-equipped personnel to a dedicated CT scanner room equipped for contagious disease within the radiology department, during dedicated hours of the day. LDCT structured report and scores were used to quantify the extent of lung abnormalities [24]. Among these LDCT, 30% were normal, 45% were minimal, 20% intermediate and 5% severe [27]. LDCT Scanner was used for follow-up. We realized 7918 genomes among them 7370 (93%) we analyzed and allow to identify 10 genetically distinct genomic variant some responsible for unique outbreak others for pandemic spread (Figure 2). We inoculated 7643, SARS-COV2 different isolates from patients in cell culture and isolated 3317 different isolates used to test susceptibility to the drug, to evaluate infectibility and help in hospital discharge management; additionally, we evaluated in vitro immune response. We realized 18,344 serology tests towards SARS Cov2 and 6520 vaccines mostly for health care workers (Table 1). We followed more than 20,000 patients, allowing description of the natural evolution of the disease (Figure 3). In terms of surge capacity, we were able with the help of the APHM to double the number of HCW and medical doctors.

The COVID-19 epidemic provided the opportunity to rapidly assess the performance of new diagnostic assays and tests, given the large amount of well-preserved, well characterized, anonymized clinical samples in the biobank, as for antigen testing [32].

This intense activity in COVID-19 diagnosis and research in the IHU yielded a total of 21 preprint articles posted on three different platforms, including the IHU web site for preprints, and 155 published and accepted for publication as papers in 84 different journals. The current total citations are 3824 (Web of Science). Additionally, a total of 132 videos, consisting of information videos and tutorials for patients and physicians posted on the IHU YouTube channel, totaled almost 60 million views, with 394,661 followers (21 April 2021).

## 4. Discussion and Conclusions

The main identified problem during the first wave of the outbreak was the lack of Personal Protective Equipment (PPE) and PCR reactant and the extreme difficulty to get provisioned due to the national and international constraints. Thanks to private donators and to the fact that IHU MI is a private institute, we were capable to buy very quickly the needed reactant (veterinarian stock) and quickly obtained the needed PPE. The successful diagnosis of COVID-19 and treatment in the IHU relied upon: (1) leadership, (2) autonomy in decision-making with immediate, supervised enforcing of decisions, (3) previous expertise in pathogen diagnosis, (4) detournement of instruments, (5) rerouting of tests: re-using ELISA plates three times after they were appropriately washed without significant loss of technical performance, after laboratory validation. Technical autonomy will be increased by an on-going facility developing PCR primers and probes production (OligoMaker 48; OligoMaker ApS, Copenhagen, Denmark). The main pathways for success are unrestricted access to care, collection of patient medical records and regular analysis of signs and symptoms, analysis of risk factors and patient outcomes, permanent adaptation of treatment protocols, well-organized monitoring of outpatients, early care and treatment based on the most efficient and/or the least toxic available drug, and proximity of the laboratory.

## Figures and Tables

**Table 1:** Numbers of interest: IHU activity during COVID-19 pandemic in Marseille from March 5 2020 to April 26 2021.

606,313		Total PCR Test provided by IHU
424,919		Total Patients tested at IHU
204,981		Patient tested at screening facility of IHU
44,089		Total patient positive for SARS Cov2
23,390		Patients positive cared at IHU
	20,270	In the day hospital as outpatient (86.6%)
	3120	In the 75 beds as inpatient (13.4%)
24,807		EKG provided before or during hydroxychloroquine treatment
5759		Low doses CT scanner for pneumonia classification
1.7%		Total case fatality ratio
0.19%		Case fatality ratio for patient attending outpatient
7918		Genome sequenced
	7370	Genomes analyzed (93%)
7643		Patients’ sample inoculated in cell culture in the BSL3
	3317	Isolates grew from cell cultures (43.3%)
6520		vaccine doses provided
